# Case Report: *Mycobacterium abscessus* knee joint infection following herbal steam bath: successful short-course oral therapy in an immunocompetent patient

**DOI:** 10.3389/fimmu.2025.1618830

**Published:** 2025-08-13

**Authors:** Jiaqing Ye, Jiahao Hao, Cuiying Zheng, Minghui Song, Chenfeng Zhang, Weili Gao, Yumei Guo, Lijie Zhang

**Affiliations:** ^1^ Department of Laboratory Medicine, Jiaxing Hospital of Traditional Chinese Medicine Affiliated to Zhejiang Chinese Medical University, Jiaxing, China; ^2^ Department of Laboratory Medicine, Hebei Medical University Third Hospital, Shijiazhuang, China; ^3^ Hebei Key Laboratory of Intractable Pathogens, Shijiazhuang Center for Disease Control and Prevention, Shijiazhuan, China

**Keywords:** *Mycobacterium abscessus* complex (MABC), *Mycobacterium abscessus* subsp. *abscessus*, knee joint infection, nontuberculous mycobacteria, drug susceptibility testing

## Abstract

**Background:**

The *Mycobacterium abscessus* complex (MABC), a multidrug-resistant environmental mycobacterium, rarely causes joint infections, which typically involve prosthetic joints. We describe the first case of native-knee *M. abscessus* infection linked to herbal steam therapy and osteoarthritis—a previously unreported scenario, accompanied by a literature review of 20 global MABC joint infection cases (2013–2024). Our findings present an alternative approach to the therapeutic guidelines for nontuberculous mycobacteria (NTM) infections, demonstrating successful clinical resolution in this single case using a short-course oral regimen.

**Case Report:**

A 54-year-old immunocompetent male with chronic knee osteoarthritis and a 6-year history of knee pain developed acute septic arthritis after knee-level high-temperature herbal steam baths. *M. abscessus* subsp*. abscessus* was identified by matrix-assisted laser desorption/ionization time-of-flight mass spectrometry (MALDI-TOF MS) and *hsp65* gene sequencing. Despite premature discontinuation of therapy, a 3-month oral regimen of clarithromycin (1,000 mg/day) combined with linezolid (600 mg/day) achieved full functional recovery, evidenced by a daily walking capacity of 8,000 steps.

**Conclusion:**

Review of 20 MABC joint infection cases from the literature revealed the knee as the most frequently affected site (55%), with the majority of patients (95%) having a history of joint surgery. This case highlights: 1) Herbal steam therapy, degenerative joint disease, and prior interventions as underrecognized risk factors; 2) Rapid molecular diagnostics (MALDI-TOF MS/*hsp65*) critical for early diagnosis; 3) Short-course oral therapy (clarithromycin/linezolid) as a potential option for localized infection when prolonged therapy is impractical.

## Background

The *Mycobacterium abscessus* complex (MABC), a group of rapidly growing *nontuberculous mycobacteria* (NTM), is widely distributed in the environment, including soil, water, and medical environments. *Mycobacterium abscessus* was first isolated in 1953 from a knee abscess in a patient and was named due to its association with subcutaneous abscess formation (1). *M. abscessus* infections have been documented in respiratory, skeletal, cutaneous, mucosal and soft tissues, though joint involvement is uncommon.

*M. abscessus* has intrinsically resistant to most anti-tuberculosis drugs and is multidrug resistant, often requiring long-term combination therapy. The treatment of *M. abscessus* infections is difficult, with a high risk of recurrence. The 2007 guidelines issued by the American Thoracic Society and Infectious Diseases Society of America (ATS/IDSA) emphasize that no single regimen guarantees a cure for *M. abscessus* infections (2). *M. abscessus* is considered to be one of the most virulent and chemotherapy-resistant RGM (3).

This case report presents a rare instance of *M. abscessus* native-knee joint infection in a patient who was treated with oral clarithromycin and linezolid, combined with joint effusion drainage. The patient demonstrated significant improvement following treatment. This report aims to enhance clinicians’ understanding of extrapulmonary NTM diseases, to improve diagnostic and therapeutic approaches.

## Case presentation

A 54-year-old man presented to our hospital on November 14, 2023, complaining of a significant exacerbation of left knee pain, against a background of a six-year history of bilateral knee pain. Over this period, he underwent various treatments, including traditional Chinese herbal medicine, massage, topical analgesic patches, acupuncture, and sodium hyaluronate injections, but none provided significant relief. One month prior to the first outpatient visit, the patient was following high-temperature of knee-level herbal steam bath for joint pain, which is a heated steam bucket containing heated water and medicinal herbs. Each therapy session lasted approximately 30 to 40 minutes. Following three days, he developed severe pain in both knees, particularly his left knee, which severely limited his ambulation. Local treatments, including analgesic patches and acupuncture at a local clinic, did not alleviate his symptoms. The patient denied a significant past medical history, including hypertension, diabetes, or prior trauma, and his general health was otherwise good.

**Figure 1:**
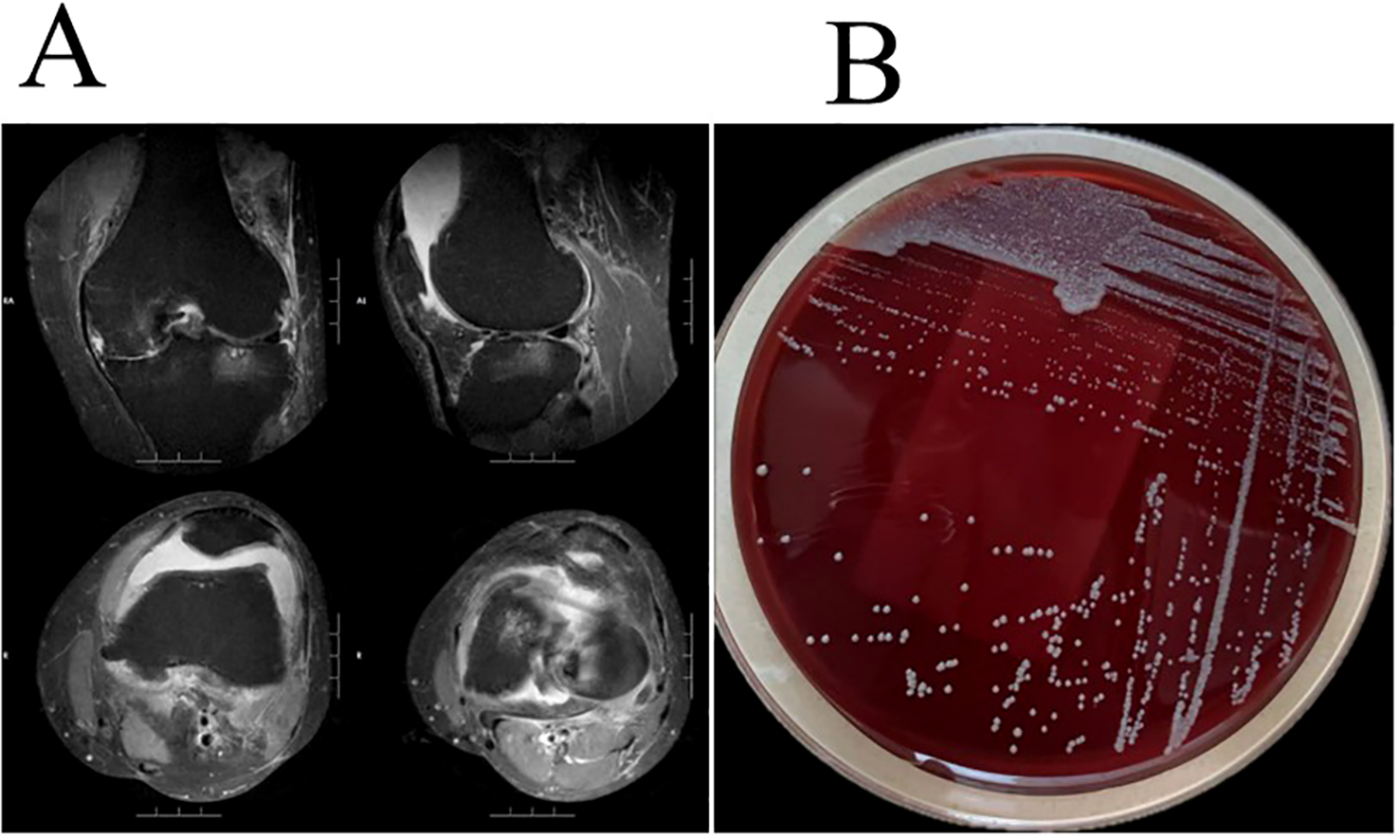
**(A)** Left knee MRI revealed osteoarthritis, a medial meniscus tear, degeneration of the lateral meniscus, and joint effusion. **(B)** *M. abscessus* colonies grown on Columbia blood agar showed moist, smooth, glossy, and circular morphology.

When the patient’s family members drove 2 hours to the outpatient clinic of the Pain Department of our hospital for the first time(Day 1), routine blood tests revealed a white blood cell count of 8.09×10^9^/L, neutrophil count of 6.44×10^9^/L, platelet count of 298×10^9^/L, and C-reactive protein (CRP) level of 17.37 mg/L. MRI of the left knee revealed osteoarthritis, a medial meniscus tear, degeneration of the lateral meniscus, and joint effusion (**Figure 1A**). The first synovial fluid sample obtained at the initial outpatient presentation (Day 1, November 14, 2023) yielded growth of the organism on Day 5(November 18, 2023). Colonies on Columbia blood agar exhibited moist, smooth, glossy, and circular morphology (**Figure 1B**). The isolate was acid-fast bacillus AFB positive and exhibited morphological characteristics highly suggestive of nontuberculous mycobacteria (NTM). Species-level identification of the isolate was achieved using matrix-assisted laser desorption/ionization time-of-flight mass spectrometry (MALDI-TOF MS) and *hsp65* sequencing. *M. abscessus* is rarely encountered in native joint infections. Given the exceptional rarity of this scenario, the patient underwent repeat joint aspiration (arthrocentesis) on Day 8 to obtain a second synovial fluid sample for culture. This was performed to rule out specimen contamination and confirm the pathogenic role of M. abscessus subsp. abscessus. The second sample collected during the second outpatient presentation (Day 8, November 21, 2023) was confirmed positive on Day 12(November 25, 2023). Collectively, the consistent isolation of *M. abscessus* subsp*. abscessus* from two separate synovial fluid cultures confirmed its role as the causative pathogen. The final diagnosis was septic arthritis of the knee caused by *M. abscessus* subsp*. Abscessus*, an atypical and rapidly growing mycobacterium.

The patient presented with bilateral knee pain, with severe pain in the left knee. His medical history revealed a six-year history of knee pain. Tendinitis and arthritis caused by tissue damage such as tears and degeneration are the most common predisposing factors for musculoskeletal NTM infections (4). This patient presented with high-risk factors for joint infection, including left knee osteoarthritis, a medial meniscus tear, and degeneration of the lateral meniscus. Laboratory findings revealed elevated neutrophil percentage and count, as well as increased C-reactive protein (CRP) levels. To identify the causative pathogen, two microbial cultures of the synovial fluid were performed, both of which yielded the same result and confirmed *M. abscessus* subsp*. abscessus* as the causative agent of the knee joint infection.

**Figure 2:**
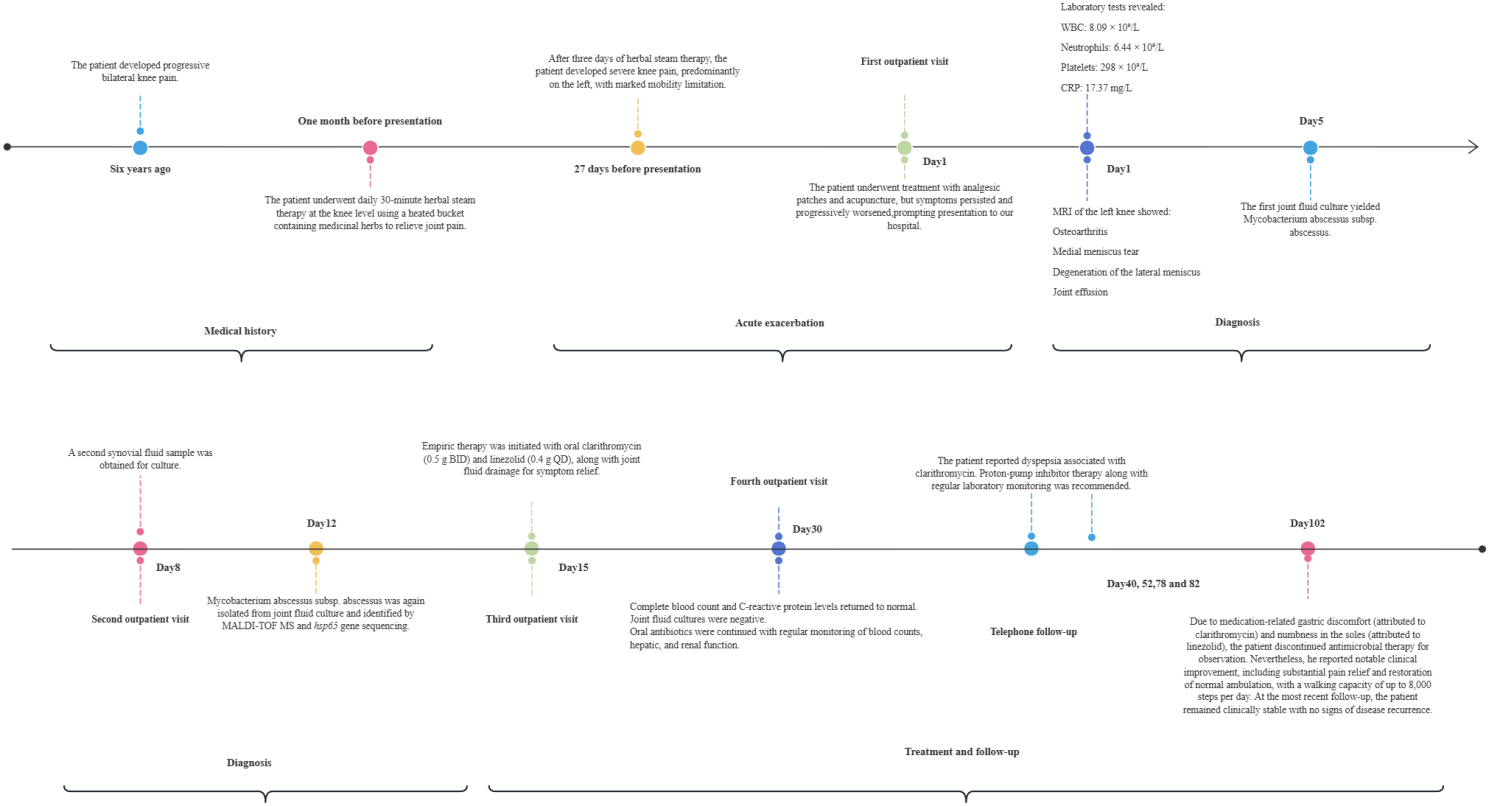
Chronological timeline summarizing the patient’s clinical course.

The patient, with a six-year history of bilateral knee pain, presented to our hospital with worsening knee pain that was more severe in the left knee. Given this asymmetric clinical presentation and pathogen localization, MRI and pathogen culture were performed only on the left knee to establish the diagnosis. Systemic antibiotic therapy combined with drainage of the left knee infection provided comprehensive coverage for potential bilateral involvement. When the patient visited the outpatient for the third time (Day 15), empirical therapy for *M. abscessus* subsp*. abscessus* infection was initiated, consisting of oral clarithromycin 500 mg twice daily and linezolid 400 mg once daily, combined with symptomatic treatments such as joint effusion drainage. The 14-day interval between initial sampling and treatment reflects the required microbial growth cycle (typically 3–4 days per culture iteration) combined with necessary logistical coordination, underscoring our caution in diagnosing this rare entity. Subsequent antimicrobial susceptibility testing showed susceptibility to clarithromycin, linezolid, cefoxitin, amikacin, and moxifloxacin. However, despite amikacin susceptibility, intravenous administration was deemed unfeasible due to insurmountable patient-specific barriers: 1) Geographic/logistical constraints (residence in another province requiring a 2-hour commute per visit, precluding daily attendance); 2) Significant caregiver burden (the patient was the primary caregiver for his wife with breast cancer, necessitating outpatient management as he explicitly declined hospitalization); 3) Outpatient safety concerns (daily IV amikacin mandates rigorous monitoring for nephrotoxicity and ototoxicity, which was impractical in this setting). Two weeks after treatment initiation (Day 30), the patient returned to our hospital for a follow-up evaluation. Routine blood tests, CRP levels were within normal limits. Joint fluid smear microscopy and bacterial cultures were negative. Crucially, joint fluid smear and culture were negative, indicating rapid microbiological conversion attributed to the synergistic effect of clarithromycin-linezolid therapy and prior therapeutic drainage (which reduced bacterial burden), directly confirming early therapeutic efficacy in this immunocompetent patient. The patient was instructed to continue oral clarithromycin and linezolid and to undergo regular monitoring of Complete Blood Counts, liver and kidney function. During the sequential telephone clinical consultations (Days 40, 52, 78, 82), the patient reported clarithromycin-associated dyspepsia. We recommended proton-pump inhibitor therapy with regular laboratory monitoring. At the Day 102 follow-up, the patient reported having consulted infectious disease specialists at Beijing Jishuitan Hospital for medication-associated gastric discomfort (caused by clarithromycin) and numbness in the soles of the feet (caused by linezolid). Based on their recommendation, he discontinued antimicrobial therapy for observation. Despite this, the patient reported significant clinical improvement, with significant pain relief and the ability to walk normally, ambulatory capacity reaching 8,000 steps/day. At the most recent follow-up, the patient remains clinically stable with no evidence of disease recurrence. The clinical timeline is summarized in **Figure 2**.

## Discussion

*M. abscessus* most commonly causes pulmonary infections and localized skin or soft tissue infections, while musculoskeletal infections involving joints are relatively rare (4, 5). When *Mycobacterium abscessus* causes joint infections, it typically infects prostheses. Skin and soft tissue NTM infections often result from trauma, surgery, cosmetic procedures, injection therapies (e.g., intralesional steroid injections or acupuncture), or other percutaneous procedures (e.g., tattooing, acupuncture and body piercings) (6, 7). Clinical manifestations of *M. abscessus* joint infections include localized pain, swelling, purulent discharge, ulceration, and draining sinuses (8, 9). However, these presentations are similar to other bacterial joint infections and lack specificity, making it difficult to distinguish the pathogen based on clinical symptoms alone. Following the taxonomic reclassification of *M. abscessus* complex (previously classified as *Mycobacterium chelonae*), a comprehensive PubMed database search (2013–2025) identified 20 cases of *M. abscessus* joint infections (**Table 1**) (10–26). Demographics revealed male predominance (12/20, 60%) over females (8/20, 40%). The most frequently affected joints were the knee (11 cases, 55%) and hip (6 cases, 30%). Concomitant bloodstream infections were observed in 4 patients (20%). Regarding treatment modalities, surgical intervention was implemented in 19 patients (95%), including 11 cases (55%) undergoing arthroplasty. Antibiotic therapy was administered to all patients, supplemented by joint debridement in 15 cases (75%). At follow-up, 16 patients (80%) demonstrated favorable outcomes.

**Table 1:** A literature review (2013–2025) identified 20 reported cases of *M. abscessus* joint infections in Pubmed.

Reference	Country/Age/gender	Infected area	Anamnesis	Surgery performed	Antibiotic	Outcome
Ng SW et al., 2015 (10)	Singapore/22-year-old/man	Knee joint	Arthroscopic right anterior cruciate ligament reconstruction with hamstring grafts	Arthroscopic washout of his right knee, Synovectomy, and Debridement of the right tibial	Amikacin, Cefoxitin and Clarithromycin	Improved
Fukui S et al., 2015 (11)	Japanese/75-year-old/woman	Right elbow joint and blood	The treatment of antisynthetase syndrome with corticosteroids, in the presence of anti-threonyl-tRNA synthetase antibodies, has been ongoing for 17 years	Surgical debridement	Clarithromycin, Amikacin, and Imipenem/Cilastatin	dead
Henry MW et al.2016 (12)	American/65-year-old/man	Hip joint	Bilateral hip replacement	Prosthesis removal with implantation of an antibiotic-loaded bone cement spacer, Prosthesis revision surgery and Open debridement	Imipenem, Amikacin and Azithromycin	Improved
Kim M et al.2017 (13)	South Korea/83-year-old/woman	Knee joint	Right primary total knee arthroplasty	Open debridement	Amikacin, Cefoxitin and Azithromycin	Improved
Kandhari VK et al.2017 (14)	India/76-year-old/woman	Knee joint	Hemimandibulectomy of the left side for oral cance	Bilateral total knee arthroplasty	Clarithromycin, Linezolid and Faropenem	Improved
Gupta N et al., 2017 (15)	India/26-year-old/man	Left femur, Hip joint and Knee joint	CD4 lymphocytopenia, Left middle cerebral artery infarct	Debridement	Imipenem, Amikacin and Macrolide	Improved
Spanyer JM et al.2018 (16)	American/61-year-old/woman	Knee joint	NA	Debridement, Synovectomy, Prosthesis Removal, Cement Spacer Implantation, Prosthesis Revision Surgery	Imipenem, Clarithromycin, Amikacin, Linezolid and Tigecycline	Improved
Amit P et al.2017 (17)	India/65-year-old/man	Knee joint	Hypertension	Prosthesis removal and Debridement	Clarithromycin, Levofloxacin and Imipenem	dead
Pace V et al., 2019 (18)	Italy/75-year-old/woman	Hip joint	Hypercholesterolemia, Gastritis and Aortic Valve Replacement	Prosthesis removal with implantation of an antibiotic-loaded bone cement spacer, Prosthesis revision surgery	Amikacin, Imipenem, and Clarithromycin	Improved
Nengue L et al., 2019 (19)	American/82-year-old/man	Knee joint	Hypertension	Right total knee arthroplasty, Debridement	Tigecycline, Amikacin and Azithromycin	Improved
Malhotra R et al.2019 (20)	India/78-year-old/man	Knee joint	Diabetic and Hypertensive	Debridement of both knees, Removal of prostheses from both knees with implantation of antibiotic-loaded bone cement spacers, Debridement of the right knee with arthrodesis, Debridement of the left knee with implantation of an antibiotic-loaded bone cement spacer, Revision of the left knee prosthesis	Rifabutin, Clarithromycin and Amikacin	Improved
Serling-Boyd N et al., 2020 (21)	Boston/80-year-old/man	Shoulder joint, Hip joint and Blood	Atrial fibrillation+ Hypertension+ Dementia+ Monoclonal Gammopathy+ Diabetes+ Rheumatoid Arthritis	Osteoarthritis status post bilateral total shoulder arthroplasty and Right hip arthroplasty	Tigecycline, Imipenem and Azithromycin	Improved
Tsuruyama Y et al., 2021 (22)	Japan/74-year-old/man	Knee joint	Diabetic nephropathy	Total knee arthroplasty	Amikacin, Imipenem/Cilastatin and Azithromycin	Improved
Genovese N et al.2021 (23)	American/79-year-old/man	Hip joint	B-cell Lymphoma, Diabetes mellitus, Hypertension B	Left hip hemiarthro plasty, Debridement	Ami Kacin, Colistin, Tigecycline, Azithromycin, Fluconazole, Eravacycline, Ceftaroline and Ceftazidime-Avibactam	Improved
Singh D et al.2022 (24)	Ethiopian/72-year-old/woman	Hip joint	Type 2 diabetes mellitus, Malnutrition and Depression	Left hip hemiarthroplasty, Debridement, Revision the antibiotic spacer, Cerclage fixation of a periprosthetic femur fracture	Tobramycin and Clarithromycin	worse
Al Mamari A et al., 2022 (25)	Oman/63-year-old/man	Knee joint and blood	Type 2 Diabetes, Hypertension and Dyslipidaemia	Knee arthroscopic debridement and Lavage	Amikacin, Tigecycline, Linezolid and Azithromycin	Improved
Aier S et al., 2023 (26)	India/20-year-old/man	Knee joint	Right knee insta bility for 2years following a history of a fall from a height	Anterior cruciate Ligament reconstruc tion, Meniscectomy, Quadrupled Hamstring graft, Surgical debridement	Amikacin, Alongside, Clarithromycin and Doxycycline	Improved
Watanabe C et al.2024 (27)	Japan/52-year-old/man	Shoulder joint and blood	Diffuse large B cell lymphoma of the primary left iliac	NA	Amikacin, Azithromycin, and Imipenem/Cilastatin.	dead
Kim JH et al., 2024 (28)	South Korea/81-year-old/woman	Elbow joint	Repetitive acupuncture therapy, *M. abscessus* pulmonary infection	Open synovectomy, Debridement	Imipenem, Azithromycin and Clofazimine	Improved

*M. abscessus* exhibits resistance to high concentrations of chlorine, organic mercury compounds, and disinfectants such as alkaline glutaraldehyde. It is capable of forming biofilms, withstanding high temperatures, and is widely distributed in various environments, including soil, tap water, showers, heating systems and medical environments (2, 27, 28). In the present case, the patient with chronic knee osteoarthritis and a 6-year history of knee pain, had undergone knee-related interventions including acupuncture and intra-articular sodium hyaluronate injections. He denied any significant trauma history and history of arthroscopic procedures or local corticosteroid injections. Subsequently, exposure to high-temperature herbal foot baths (using self-cultivated mugwort) that immersed the knees occurred, potentially representing a plausible environmental source for *M. abscessus*. High-temperature herbal fumigation therapy, pre-existing degenerative joint disease, and invasive procedures may have compromised the integrity of the skin barrier, thus providing a pathway for *M. abscessus* subsp*. abscessus* to invade the joint cavity and ultimately cause acute infectious arthritis.

MABC consists of three subspecies: *M. abscessus* subsp. *abscessus*, *Mycobacterium abscessus* subsp. *massiliense*, and *Mycobacterium abscessus* subsp. *bolletii*. Antimicrobial susceptibility varies between and within these subspecies (2), necessitating precise subspecies-level identification and comprehensive antimicrobial susceptibility testing for all clinically relevant isolates. Monotherapy poses substantial challenges; consequently, current clinical guidelines advocate for combination therapy involving at least 3–4 antimicrobial agents (29). However, the optimal combination of oral and intravenous agents remains undefined (30). For severe bone and soft tissue infections caused by *M. abscessus*, oral macrolides combined with intravenous medications (e.g., amikacin, cefoxitin, or imipenem) are commonly recommended (20). According to expert consensus, recommended intravenous medications such as amikacin (10–15 mg/kg/day or 15/25 mg/kg three times weekly), imipenem (1 g two to three times daily), cefoxitin (6–8 g/day in three to four divided doses), and tigecycline (25–50 mg once or twice daily), as well as oral agents such as azithromycin (250–500 mg/day or 500 mg three times weekly), clarithromycin (500 mg twice daily), clofazimine (100 mg/day), linezolid (600 mg/day), and newer drugs like tedizolid (200 mg/day), omadacycline (300 mg/day), and bedaquiline (400 mg/day for 14 days, then 200 mg three times weekly) (29). A recent study revealed notably diminished intracellular accumulation of linezolid, implying that restricted cellular penetration may limit its antimicrobial activity. As a ribosome-targeting agent, linezolid exerts its bacteriostatic effect through binding to the 23S rRNA component of the 50S ribosomal subunit. While this oxazolidinone derivative forms part of combination therapies against multidrug-resistant *Mycobacterium tuberculosis* and various Gram-positive pathogens, its standalone efficacy against *M. abscessus* in liquid culture is limited. Nevertheless, emerging evidence supports its therapeutic potential as an adjunctive agent, demonstrating synergistic interactions with first-line anti-mycobacterial agents such as amikacin and clarithromycin. Clinical observations correlating linezolid-containing regimens with improved patient outcomes further reinforce this perspective. Intriguingly, unlike resistance mechanisms documented in other bacterial species, *M. abscessus* clinical isolates rarely exhibit ribosomal target site mutations despite prolonged therapeutic exposure. This phenomenon may be partially explained by upregulated efflux pump activity observed in resistant strains, coupled with experimental evidence of mutations in efflux-related essential genes during *in vitro* selection. These findings collectively suggest that enhancing intracellular drug retention could optimize linezolid’s clinical utility against this pathogen (31).

In this case, empirical therapy with oral clarithromycin and linezolid was initiated. Antimicrobial susceptibility testing subsequently confirmed susceptibility to both agents, supporting continuation of this regimen. Due to the patient’s role as sole caregiver for his spouse with breast cancer, treatment was administered exclusively in the outpatient setting. Clinical management required only four outpatient visits for diagnosis, therapeutic planning, and efficacy assessment.

For patients with extensive lesions, abscess formation, or poor response to pharmacological therapy, surgical intervention including active debridement or removal of foreign bodies is recommended. The recommended treatment duration for bone and joint NTM infections is 6 to 12 months (32). Notably, even after complete debridement and prolonged appropriate antimicrobial therapy, infections may recur, necessitating repeated surgical interventions (33, 34).

In the present case, the patient self-discontinued medication after nearly three months due to drug adverse effects, including gastric discomfort (attributed to clarithromycin) and numbness in the soles of the feet (attributed to linezolid). It is important to acknowledge that this premature discontinuation, falling significantly short of the guideline-recommended 6 to 12 months for bone and joint NTM infections, constitutes a significant risk factor for disease recurrence. Despite this risk, follow-up assessments to date have shown sustained clinical recovery, with restored ability to walk normally and a daily step count of approximately 8,000 steps. At the most recent follow-up, no recurrence was confirmed, suggesting that some patients may be treated with a shorter course of treatment for MABC knee joint infection. While this case provides preliminary evidence that an abbreviated therapeutic regimen may be achievable in comparable scenarios, this shorter-course approach requires rigorous validation through larger prospective multicenter studies.

## Data Availability

The original contributions presented in the study are included in the article/supplementary material. Further inquiries can be directed to the corresponding author.
